# Supplementary figures and images for: Enhancing Triage Efficiency and Accuracy in Emergency Rooms for Patients with Metastatic Prostate Cancer: A Retrospective Analysis of Artificial Intelligence-Assisted Triage Using ChatGPT 4.0
Source: Cancers (Basel). 2023 Jul 22;15(14):3717. doi: 10.3390/cancers15143717 (PMC10378202; doi:10.3390/cancers15143717)

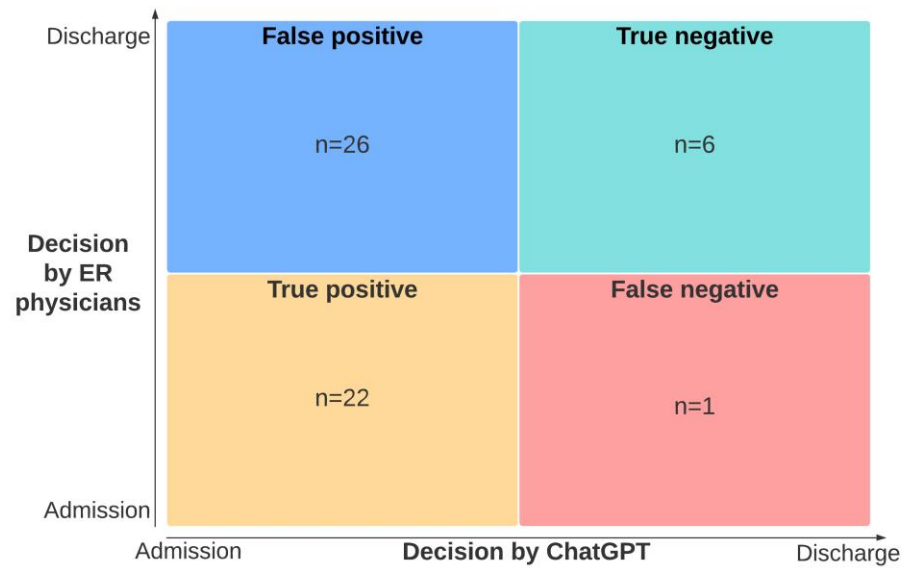

*Figure S1.* Confusion matrix between ER physicians and ChatGPT.

Supplement: Supplementary file 1 [file cancers-15-03717-s001.zip › cancers-2500650-supplementary.pdf]
